# Establishment of a novel protocol for formalin-fixed paraffin-embedded organoids and spheroids

**DOI:** 10.1242/bio.059882

**Published:** 2023-05-16

**Authors:** Shohei Yoshimoto, Masahide Taguchi, Satoko Sumi, Kyoko Oka, Kazuhiko Okamura

**Affiliations:** ^1^ Section of Pathology, Department of Morphological Biology, Division of Biomedical Sciences, Fukuoka Dental College, Fukuoka 814-0193, Japan; ^2^ Oral Medicine Research Center, Fukuoka Dental College, Fukuoka 814-0193, Japan; ^3^ Section of Pediatric Dentistry, Department of Oral Growth and Development, Fukuoka Dental College, Fukuoka 814-0193, Japan

**Keywords:** Formalin-fixed paraffin-embedded (FFPE), Organoid, Spheroid, Immunohistochemistry, Three-dimensional experimental model

## Abstract

Three-dimensional (3D) cell culture models such as spheroids and organoids are widely used in the field of experimental biology. To analyze these 3D experimental models, formalin-fixed paraffin-embedded (FFPE) sections are superior to whole-mount imaging for some experimental purposes, such as exploring samples with a depth limitation of primary antibody penetration immunohistochemically. However, tiny 3D cell culture samples are difficult to embed in paraffin and acquire appropriate sections. In this report, we optimized a protocol of paraffin embedding for spheroids and organoids. In addition, we compared FFPE sections with frozen sections in ratio of sample collection and section condition after staining, and could reproduce improved results reliably. The protocol we established could be widely used in many laboratories and become a useful technique for analyzing spheroids and organoids.

## Teaser

**Summary:** Three-dimensional cell culture models such as spheroids and organoids are used in the field of experimental biology. This paper presents an optimized protocol of paraffin embedding for spheroids and organoids.

## INTRODUCTION

In life science studies, using three-dimensional (3D) cell culture models has enabled various physiological and pathological experiments resembling *in vivo* conditions to be performed (Fatehullah et al., 2016; Feng et al., 2009; Pringle et al., 2016). It is well known that *in vivo* conditions, such as RNA and protein expression, can be much better reflected in the 3D experimental models than in the conventional two-dimensional (2D) cell culture models. In the 3D experimental models, spheroid and organoid culture models are widely used (Sato et al., 2009, 2011; Yoshimoto et al., 2022). To analyze these models, imaging techniques, such as whole-mount immunofluorescence, are often selected. However, these techniques have some limitations in the number of staining targets on the same samples. For some experimental purposes, histochemical analysis by serial sectioning is often required.

Paraffin embedding is a widely used technique in clinical pathology and experimental histopathology. Formalin-fixed paraffin-embedded (FFPE) samples are easy to handle when we want to slice serial sections. In this report, we describe an optimized a protocol of paraffin embedding for spheroids and organoids. Moreover, we compared FFPE sections with frozen sections in ratio of samples collection and section condition.

## RESULTS

### Protocol for FFPE organoids and spheroids

**Fig. 1.**
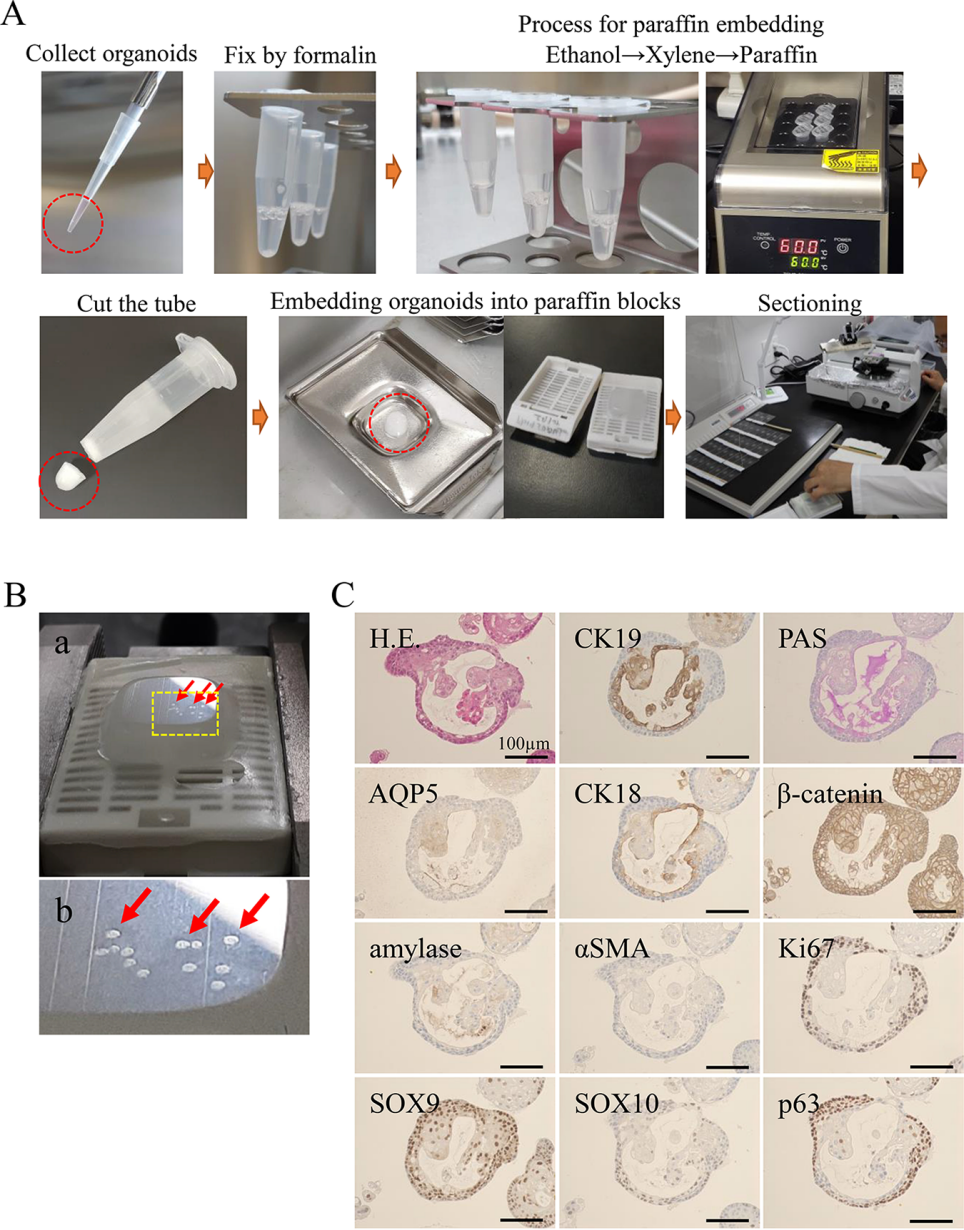
**Protocol for FFPE organoids and spheroids.** (A) Protocol for FFPE organoids and spheroids. Photographs of each step are shown in order. The tip of tube of the embedding mold is circled by a red dotted line. (B) Spheroids (red arrows) in an FFPE block during the sectioning process (a); a magnified image is indicated in the lower panel (b). (C) Panels of salivary-gland-derived organoids. HE, PAS and immunohistochemical staining using each antibody. Scale bar: 100 μm.

First, we present the protocol for FFPE organoids and spheroids as follows: (1) Collect organoids in 1.5 ml centrifuge tube by centrifugation at 400×***g*** for 5 min. Remove the medium and wash the organoids with ice-cold, sterile 1× phosphate-buffered saline (PBS). Centrifuge at 400×***g*** for 5 min to collect the organoids. Discard the supernatant. Visually inspect the samples and carefully pipette the supernatant in each step. If the size of the samples is >100 μm, there is less chance of the samples being lost. (2) Add 1 ml 10% neutral buffered formalin (or 4% paraformaldehyde) fixative, and fix organoids for 30 min at room temperature. Centrifuge at 400×***g*** for 5 min to remove fixatives (Fig. 1A). (3) The process for paraffin embedding schedule is as follows: (a) 70% ethanol for 30 min; 80% ethanol for 30 min; 90% ethanol for 30 min; 100% ethanol for 30 min; xylene for 1 h, twice; paraffin wax (60°C) for 2 h on dry baths or heat blocks (Fig. 1A). These processes are performed in the 1.5 ml centrifuge tube. Centrifuge at 400×***g*** for 5 min to remove ethanol and xylene. Then cut at 5 mm from the bottom of the tube containing organoids. Put the tip of tube on the embedding mold and melt paraffin wax. Next, melt the wax, move organoids into the embedding mold. Embedding organoids into paraffin blocks (Fig. 1A). In this embedding step, if the samples stick to the bottom of the tube, remove them and embed them with forceps carefully. (4) Trim paraffin blocks and cut at 4 µm. Serial sections can be obtained from an organoid. We can easily find FFPE organoids or spheroids during sectioning (Fig. 1B). (5) Mount sections on slides and bake them on 50°C hotplate overnight. If sections are applied for immunostaining with antigen-retrieval, the use of adhesive glass slides with some coating or treatment is recommended to prevent the loss of spheroid sections. (6) Deparaffinize sections in xylene for 5 min, twice. (7) Hydrate in 100% ethanol for 3 min, twice, then in 95%, 80% and 70% ethanol for 1 min each. (f) Proceed to staining.

The advantages of our FFPE method are that the process is completed in a single 1.5 ml centrifuge tube, samples are easily stored, and serial sections can be easily made from a small organoid. Serial sections are available for Hematoxylin and Eosin (HE) staining, special staining such as Periodic acid–Schiff (PAS) staining and immunostaining (Fig. 1C). Analysis using human salivary gland organoids showed that it is possible to examine the expression of numerous salivary-gland-related protein markers from the same organoid.

### Comparison of FFPE sections with frozen sections

**Fig. 2.**
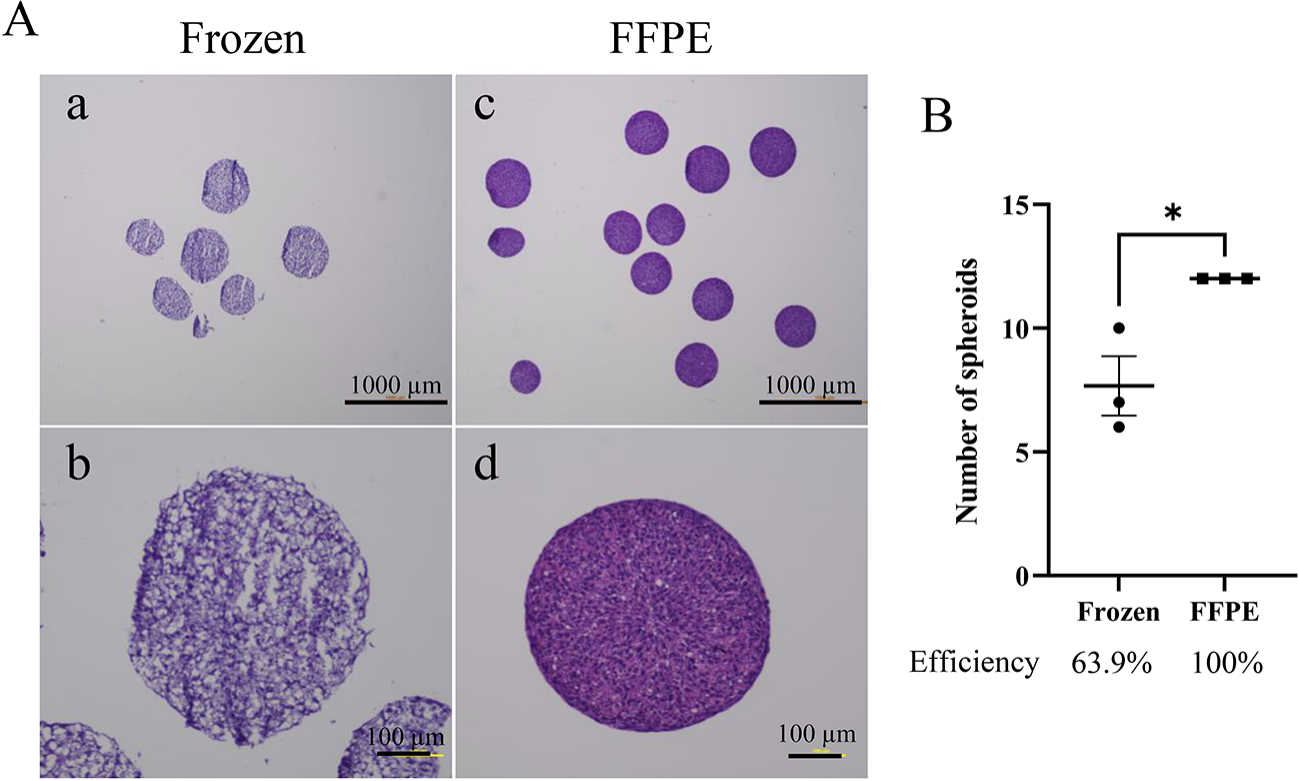
**Comparison of FFPE sections with frozen sections.** (A) HE staining of HSC-4 cell spheroids in a frozen section (a,b) and in a FFPE section (c,d). Magnified images are indicated in the lower panels (b,d). Scale bar: 1000 μm (a,c); 100 μm (b,d). (B) Graph indicating the number of observable spheroids in the sections. Statistical significance was set as *P<0.05 (*n*=3).

The frozen section procedure is also widely used in many laboratories for microscopic analysis of a specimen. We compared the quality of FFPE sections with frozen sections in HE staining. In frozen sections of spheroids, some crushing of the samples was observed and details of individual cells were not clear. Whereas almost all spheroids retained their round shape, and cell–cell contact and the shape of each cell were maintained in FFPE sections (Fig. 2A). We next examined the observation efficiency of both methods. We prepared twelve spheroids per each group, and performed FFPE or frozen section procedures. After sectioning and staining, spheroids appearing on the same sections were counted. In frozen sections, the efficiency of observable spheroids was 63.9%. In FFPE sections, the efficiency of observable spheroids was 100%. A significant upregulation of the efficiency was found in the FFPE method (*P*=0.0226, Fig. 2B). Compared with frozen sections, FFPE sections were more physically stable and showed excellent tissue morphology and efficiency of observation.

## DISCUSSION

In this paper, we present a protocol of paraffin embedding for spheroids and organoids. Generally, an automated tissue processor is used for paraffin embedding in the pathology laboratory. However, spheroids and organoids are too small to be processed in this machine. Our paraffin embedding protocol using centrifuge tubes could process small samples without loss. Moreover, the equipment needed for this protocol is widely available in many laboratories.

One advantage of FFPE sections is their compatibility with HE staining. HE staining is suitable for morphological observation. The frozen section technique is commonly used in clinical pathology for rapid microscopic diagnosis. However, it is well known that the quality of the slides of frozen sections is lower than that of FFPE sections. In our observation, frozen spheroids showed mildly collapsed, rough and shaggy shapes in the sections (Fig. 2A). Thus, our results showed that FFPE sections were more physically stable and showed excellent tissue morphology. Moreover, to obtain the sections with appropriate direction for observation, we needed to make serial sections and HE staining at a specific intervals. The FFPE method is more suitable for serial sectioning and preservation than frozen methods.

Our verification showed that the rate of sample collection, caused by that the samples are in the same plane and appear in the same section, was higher with the FFPE method than with the frozen method (Fig. 2B). Our protocol also has advantages over frozen sections in terms of reproducibility and less variability in results. In frozen section procedure, samples were not stable during embedding in compound. On the other hand, in the FFPE method, it was easy to embed samples at the same level. This ease of embedding was an advantage of the method. Thus, the efficiency of observable samples in FFPE sections was higher than that of the frozen method. Another advantage of FFPE is that it can be stored stably for long periods at room temperature. It would be possible to construct an organoid or spheroid library. In contrast, most of the antibodies require antigen-retrieval steps in FFPE sections. In some cases, depending on the antibodies, frozen sections are required for immunofluorescence. Whole-mount immunofluorescence is a better technique than sectioning for obtaining 3D information. It would be important to use appropriate experimental techniques depending on the purpose. This method could be widely used in many laboratories and become a useful technique for analyzing spheroids and organoids.

## MATERIALS AND METHODS

### Organoid and spheroid culture

Methods of salivary gland organoid culture have been previously described (Yoshimoto et al., 2020). This study was performed with the permission of the ethics committee in Fukuoka Dental College (number 406). Salivary glands were obtained from the patients with mucous cysts or head and neck tumors who underwent an operation in Fukuoka Dental College hospital. Human squamous cell carcinoma cell line, HSC-4, was purchased from JCRB cell bank (Osaka, Japan). Spheroid culture was previously described (Yoshimoto et al., 2022, 2023). Briefly, 1×10^4^ cells in 200 μl Dulbecco's Modified Eagle's Medium (DMEM, Sigma-Aldrich, St. Loius, MO, USA) with 10% FBS were seeded in each well of 96-well Nunclon Sphera plate (Thermo Fisher Scientific, Waltham, MA, USA). 10% neutral buffered formalin or 4% paraformaldehyde -fixed and paraffin-embedded organoids tissue blocks were cut into 4-μm-thick sections and mounted on adhesive glass slides (CRE-01, Matsunami Glass, Osaka, Japan) for HE and immunohistochemical staining. Frozen sections were prepared by embedding samples in Tissue-Tek optimal-cutting-temperature (OCT) compound (Sakura Finetek Japan, Tokyo, Japan) and cryosectioning. The images of HE and immunohistochemical staining were captured using an AXIO Vert.A1 microscope (Carl Zeiss, Oberkochen, Germany).

### Antibodies

The primary antibodies; AQP5 (1:500 dilution; #AQP-005, Alomone labs, Jerusalem, Israel), CK18 (1:100; Clone DC 10, DAKO-Agilent Technologies, Santa Clara, CA, USA), p63 (1:100; Clone 4A4, DAKO), α-Smooth Muscle Actin (1:100; Clone 1A4, DAKO), CK19 (1:100; Clone A-3, Santa Cruz Biotechnology, Santa Cruz, CA, USA), α-amylase (1:200; Clone G-10, Santa Cruz Biotechnology), SOX9 (1:100; #AB5535, Millipore, Burlington, MA, USA), SOX10 (1:100; Clone A-2, Santa Cruz Biotechnology), β-Catenin (1:100; #610154, BD Biosciences, Franklin Lakes, NJ, USA), Ki67 (1:50; Clone MM1, Leica Biosystems, Deer Park, IL, USA). The secondary antibodies; horseradish peroxidase (HRP)-conjugated polymer anti-rabbit and -mouse antibodies were purchased from DAKO-Agilent Technologies.

### Statistical analysis

All data were expressed as the mean±standard error of the mean (s.e.m.). A two-tailed Student's *t*-test was used for statistical evaluations. Statistical significance was set as *P<0.05.
